# Generalized anxiety and loneliness among the general population in Saudi Arabia

**DOI:** 10.1186/s12888-026-08080-3

**Published:** 2026-04-17

**Authors:** Rehab Alhazmi, Riyam Alhazmi

**Affiliations:** 1 King Abdullah International Medical Research Center, Riyadh, Saudi Arabia; 2 College of Nursing, King Saud bin Abdulaziz University for Health Sciences, Riyadh, Saudi Arabia; 3 Ministry of the National Guard - Health Affairs, Riyadh, Saudi Arabia; 4 Outpatient Psychiatric Pharmacy, Dr. Erfan and Bagedo General Hospital, Jeddah, Saudi Arabia

**Keywords:** Loneliness, Anxiety, Saudi Arabia, Population, Adults

## Abstract

**Background:**

Loneliness has gained increasing attention for its detrimental effects on individuals’ mental and physical health. Anxiety, among other psychiatric conditions, has been associated with loneliness. This study assessed the association between loneliness and generalized anxiety and explored potential social moderators of this relationship. Furthermore, it aimed to identify sociodemographic and social factors associated with loneliness and generalized anxiety.

**Methods:**

This cross-sectional study included 1084 adults aged ≥ 18 years. Participants completed a sociodemographic and social factors questionnaire, the Generalized Anxiety Disorder-7 (GAD-7), and the three-item University of California, Los Angeles (UCLA) Loneliness Scale. Bivariate analyses were performed to assess factors associated with loneliness and generalized anxiety. In addition, moderation analyses were conducted to examine potential social factors moderating the association between loneliness and generalized anxiety.

**Results:**

The findings revealed that loneliness had a significant positive association with generalized anxiety (*p* < 0.05). Both the frequency of contact with others and having someone to rely on and discuss problems with moderated this association. Individuals who lived alone; did not have someone to rely on and discuss problems with; had a personal history of psychiatric consultation or medication use; and had less frequent contact with friends, family members, and neighbors exhibited higher mean scores for loneliness and generalized anxiety compared with their counterparts. Furthermore, individuals with chronic disorders exhibited higher mean loneliness scores. Older age was significantly associated with lower generalized anxiety scores.

**Conclusions:**

These findings highlight the detrimental effect of loneliness on mental health. Having someone to rely on and discuss problems with, as well as frequent contact with friends, family members, and neighbors, can buffer the negative impact of loneliness on mental health. Therefore, initiating appropriate strategies and public health policies to reduce loneliness and mitigate its negative consequences is necessary. These findings provide valuable insights for future research, particularly in Saudi Arabia, on potential moderators in the relationship between loneliness and anxiety.

**Clinical trial number:**

Not applicable.

## Background

The influence of loneliness on individuals’ mental and physical health is an increasing public health concern [1, 2]. A recent review identified a link between loneliness and several psychiatric and physical health conditions [3]. Its prevalence is reported to be 10.5–48.2% across different countries [4–6]. Loneliness is described as individuals’ dissatisfaction with the quality and quantity of social contacts they engage in or desire [7, 8]. One may experience feelings of being alone even when surrounded by multiple people [7]. Loneliness is a perceived social isolation, a distinct psychological construct that differs from objective social isolation [7, 8].

Research has associated loneliness with various demographic factors. The risk of loneliness decreases among individuals with higher education, those who are married, or those who have children [1, 9]; conversely, the risk increases among those who are unemployed, have low financial status [9], or live in urban areas [10]. Furthermore, loneliness is common among young people and older adults but less prevalent among middle-aged adults [7, 9]. Evidence regarding gender shows contradictory results; some studies have reported that women are more likely to experience feelings of loneliness than men [4], while others have reported higher loneliness among men [1]. In addition, several physical health conditions have been linked to loneliness, including metabolic disorders, cardiovascular disease, and premature mortality [1, 7, 8].

Loneliness is strongly influenced by various social factors, including living arrangements, frequency of contact with others, social support, and participation in social activities or volunteering [5, 9, 11]. Building and maintaining social connections play a crucial role in shaping feelings of safety and well-being and in promoting survival [1, 7]. Evidence strongly suggests that individuals who have strong support systems, frequent contact with family members and friends, live with others, and engage in social activities are less likely to experience loneliness [9, 12]. Therefore, understanding how these factors influence the experience of loneliness may help identify potential protective and risk factors and promote individuals’ mental health.

Extensive literature has underlined the detrimental impact of loneliness on mental health. Evidence indicates that loneliness is associated with depression, anxiety, psychosis, and suicidal ideation [4, 7, 8, 13, 14]. According to the loneliness regulatory model, individuals who experience loneliness often exhibit hypervigilance in social situations and develop related cognitive biases [7]. They may also show increased anxiety and stress sensitivity, feelings of being unsafe, impulsivity, poor self-regulation, low self-esteem, poor social skills, and engagement in unhealthy behaviors [1, 7, 15]. These negative thoughts and behaviors may persist in a self-reinforcing loneliness loop [1, 7, 16]. Previous studies found evidence for the association between anxiety and loneliness in all age groups [4, 16–18].

The impact of loneliness on mental health highlights the need to understand potential moderators of this relationship. However, limited research has examined factors that may influence this association. A recent study investigated the moderating role of social ecological factors in the relationship between loneliness and personal well-being among young adults in England and found that communication with neighbors and receiving support from others acted as moderators [11]. Another study reported that gender moderated the association between loneliness and both depression and anxiety among Norwegian adolescents [14]. In addition, one study found that social support moderated the relationship between depression and loneliness among older adults [19]. Thus, further research is needed to examine factors that may moderate the relationship between loneliness and mental health.

In Saudi Arabia, most research on loneliness and mental health has been conducted during the COVID-19 pandemic, and studies identifying factors that may moderate this relationship are scarce. Given the negative psychological impacts of loneliness, understanding this phenomenon is important for developing public health policies and effective interventions. Accordingly, this study aimed to assess the association between loneliness and generalized anxiety and to determine whether social factors—such as living arrangements; number of close friends and family members; frequency of contact with family, friends, and neighbors; participation in social activities or volunteering; and perceived social support—moderate this relationship. The study also sought to identify sociodemographic and social factors associated with loneliness and generalized anxiety.

### Research question 1

Is there an association between loneliness and generalized anxiety in the general population of Saudi Arabia?

### Research question 2

Which sociodemographic and social factors (living arrangements, number of close friends and family members, frequency of contact with others, participation in social activities or volunteering, and perceived social support) are associated with (a) loneliness and (b) generalized anxiety in the general population of Saudi Arabia?

### Research question 3

Do social factors (living arrangements, number of close friends and family members, frequency of contact with others, participation in social activities or volunteering, and perceived social support) moderate the association between loneliness and generalized anxiety in the general population of Saudi Arabia?

## Methods

### Study design and participants

This cross-sectional study included adults aged ≥ 18 years residing in Saudi Arabia. Data were collected between October 2024 and April 2025.

### Sample size and sampling technique

Raosoft was used to calculate the required sample size using the following parameters: a margin of error of 5%, a confidence level of 95%, and a distribution of 50%. Accordingly, a minimum of 385 participants was required. A convenience sample of 1084 adults was ultimately included. Electronic surveys and consent forms were distributed through social media platforms, including Telegram, WhatsApp, and X.

### Outcome measurements

Sociodemographic data included age, gender, nationality, marital status, region and area of residence, educational level, employment status, monthly income, presence of chronic disease, and personal history of psychiatric consultation or medication use. Social factors included living arrangement (living alone or with others); number of close friends and family members (≤ 2 or > 2); frequency of contact with family members, friends, and neighbors via phone calls, text messages, or in-person meetings (≤ twice or > twice per month); and participation in social activities or volunteering (yes or no). Moreover, perceived social support was assessed by asking participants whether they had someone to rely on and discuss their problems with (yes or no). Sociodemographic and social variables were developed based on the literature [5, 9, 11]. Data were collected using the Generalized Anxiety Disorder-7 (GAD-7) and the three-item University of California, Los Angeles (UCLA) Loneliness Scale.

The items of the GAD-7, a self-administered questionnaire, were rated on a 4-point Likert scale ranging from 0 (not at all) to 3 (nearly every day) [20]. Total scores range from 0 to 21, with higher scores indicating greater severity of anxiety symptoms. The GAD-7 has been widely used to assess anxiety, including in Saudi Arabia [21, 22], and has demonstrated good reliability in this context, with a reported Cronbach’s alpha of 0.76 [22]. In the present study, Cronbach’s alpha was 0.77.

The three-item UCLA Loneliness Scale was used to assess loneliness. Responses are rated on a 3-point Likert scale ranging from 1 (hardly ever) to 3 (often), yielding total scores ranging from 3 to 9, with higher scores indicating greater loneliness. The Arabic version of the scale has demonstrated good internal consistency, with a Cronbach’s alpha of 0.77 [23]. In this study, Cronbach’s alpha was 0.75.

### Statistical analysis

Statistical analyses were conducted using the Statistical Package for the Social Sciences (SPSS) version 30. Categorical variables were presented as frequencies and percentages, while continuous variables were presented as means and standard deviations (SD). Correlation analyses were used to assess associations between variables. Spearman’s correlation was used to examine associations involving ordinal variables, whereas Pearson’s correlation was used to determine associations between continuous variables. Student’s t-test was used to examine differences in mean generalized anxiety and loneliness scores across sociodemographic and social factors with two categorical groups. Moderation analyses were conducted using the PROCESS macro [24] to examine whether social factors moderated the association between loneliness and generalized anxiety. Variables with *p* < 0.05 in the bivariate analyses were included as covariates in the moderation models. Variables such as number of close friends and family members, frequency of contact with others, and living arrangement were initially recorded in multiple categories and subsequently recoded into binary variables owing to small cell counts in some categories. The level of statistical significance was set at *p* < 0.05.

## Results

### Participants’ sociodemographic, social factors, loneliness, and generalized anxiety

This study included 1084 adults from Saudi Arabia. Most participants were aged 30–39 years (34.9%, *n* = 378), Saudi Arabian (93.8%, *n* = 1017), female (67.6%, *n* = 733), not married (63.6%, *n* = 689), had a bachelor’s degree (61.3%, *n* = 664), and lived in the central region (54.2%, *n* = 587). Approximately 87.3% lived in urban areas (*n* = 946), and 45.8% had a monthly income of ≤ 10,000 Saudi riyal (SR; *n* = 497). Furthermore, 94.3% lived with others (*n* = 1022); 51.1% had ≤ 2 close friends or family members (*n* = 554); 75.8% had someone to rely on and discuss problems with (*n* = 822); 79.4% communicated > 2 times per month with friends, family, or neighbors (*n* = 861); and 60.0% volunteered in social activities in their community (*n* = 650). The mean total scores for UCLA and GAD-7 were 4.57 (SD = 1.71) and 4.44 (SD = 3.81; Table 1), respectively.

**Table 1:** Participants’ sociodemographic characteristics (*N* = 1084)

Variables	Total sample (*N* = 1084)		Saudi Arabia’s 2022 census (%)
Age n (%)			
18–29	353	32.6	31.4
30–39	378	34.9	32.1
40–49	203	18.7	19.5
50–59	85	7.8	10.4
60–69	59	5.4	4.4
≥ 70	6	0.6	2.2
Gender n (%)			
Female	733	67.6	38.8
Male	351	32.4	61.2
Nationality n (%)			
Saudi	1017	93.8	58.4
Non-Saudi	67	6.2	41.6
Marital status n (%)			
Not married	689	63.6	45.0
Married	395	36.4	55.0
Region of residence n (%)			
Eastern	83	7.7	15.9
Southern	71	6.5	13.5
Central	587	54.2	30.8
Northern	75	6.9	8.0
Western	268	24.7	31.5
Area of residence n (%)			
Urban	946	87.3	
Rural	138	12.7	
Education level n (%)			
High school and less	347	32.0	
Bachelor’s degree	664	61.3	
Master and above	73	6.7	
Employment status n (%)			
Employed	591	54.5	
Not employed	493	45.5	
Monthly income n (%)			
≤SR 10,000	497	45.8	
SR 10,001–20,000	302	27.9	
> SR 20,000	285	26.3	
Chronic disorders n (%)			
No	892	82.3	
Yes	192	17.7	
Personal history of psychiatric consultation/medication			
No	1013	93.5	
Yes	71	6.5	
Living arrangement n (%)			
Alone	62	5.7	
With others	1022	94.3	
Number of close friends and family members n (%)			
≤ 2	554	51.1	
> 2	530	48.9	
Having someone to rely on and discuss problems n (%)			
No	262	24.2	
Yes	822	75.8	
Frequency of contact with family members, friends, and neighbors per month n (%)			
≤ 2	223	20.6	
> 2	861	79.4	
Volunteering in social activity n (%)			
No	650	60.0	
Yes	434	40.0	
UCLA Mean (SD)	4.57 ± 1.71		
GAD-7 Mean (SD)	4.44 ± 3.81		

Note. Saudi Arabia’s 2022 census data were regrouped to match the study’s region, marital status, and age categories

### Bivariate analysis of factors associated with loneliness and generalized anxiety

Tables 2 and 3 present the bivariate analyses of sociodemographic and social factors associated with loneliness and generalized anxiety. Participants who lived alone; had a chronic disorder; did not have someone to rely on and discuss problems with; had a personal history of psychiatric consultation or medication use; and had less frequent contact with friends, family members, and neighbors (≤ 2 times per month) exhibited higher mean loneliness scores compared to their counterparts. Similarly, participants who lived alone, did not have someone to rely on and discuss problems with; had a personal history of psychiatric consultation or medication use; and had less frequent contact with friends, family members, and neighbors (≤ 2 times per month) exhibited higher mean generalized anxiety scores compared with their counterparts. Older age and higher loneliness scores were significantly associated with lower and higher generalized anxiety scores, respectively.

**Table 2:** Bivariate analysis of the factors associated with loneliness and generalized anxiety (*N* = 1084)

Variables	Loneliness		Generalized anxiety	
	Mean ± SD	*p*-value	Mean ± SD	*p*-value
Gender				
Female	4.59 ± 1.74	0.696	4.51 ± 3.98	0.438
Male	4.54 ± 1.65		4.31 ± 3.43	
Marital status				
Not married	4.65 ± 1.68	0.076	4.45 ± 3.73	0.995
Married	4.45 ± 1.75		4.44 ± 3.96	
Area of residence				
Urban	4.55 ± 1.69	0.321	4.45 ± 3.84	0.862
Rural	4.71 ± 1.86		4.39 ± 3.64	
Employment status				
Employed	4.58 ± 1.66	0.843	4.36 ± 3.72	0.413
Not employed	4.56 ± 1.78		4.55 ± 3.93	
Chronic disorders n (%)				
No	4.53 ± 1.69	0.047	4.39 ± 3.76	0.310
Yes	4.80 ± 1.80		4.70 ± 4.08	
Personal history of psychiatric consultation/medication				
No	4.50 ± 1.67	< 0.001	4.32 ± 3.69	< 0.001
Yes	5.64 ± 1.94		6.22 ±5.03	
Living arrangement n (%)				
Alone	5.29 ± 2.06	< 0.001	5.64 ± 5.07	0.011
With others	4.53 ± 1.68		4.37 ± 3.72	
Number of close friends and family members n (%)				
≤ 2	4.64 ± 1.79	0.215	4.51 ± 3.85	0.577
> 2	4.51 ± 1.62		4.38 ± 3.77	
Having someone to rely on and discuss problems				
No	4.94 ± 1.79	< 0.001	5.01 ± 4.07	0.006
Yes	4.46 ± 1.67		4.27 ± 3.71	
Frequency of contact with family members, friends, and neighbors per month				
≤ 2	4.93 ± 1.78	< 0.001	5.06 ± 4.35	0.007
> 2	4.48 ± 1.68		4.28 ± 3.65	
Volunteering in social activity				
No	4.60 ± 1.74	0.529	4.56 ± 4.00	0.243
Yes	4.53 ± 1.67		4.28 ±3.51	

Note. *p < 0.05* = statistically significant

**Table 3:** Correlations of the ordinal and continuous variables (*N* = 1084)

Variables	Loneliness		Generalized anxiety	
	*r*	*p*-value	*r*	*p*-value
Loneliness			0.41	< 0.001
Age	− 0.04	0.171	− 0.08	0.004
Education level	0.01	0.795	0.01	0.848
Monthly income	− 0.04	0.133	− 0.04	0.166

Note. *p < 0.05* = statistically significant

### Moderating analysis of social factors associated with loneliness and generalized anxiety

**Fig. 1:**
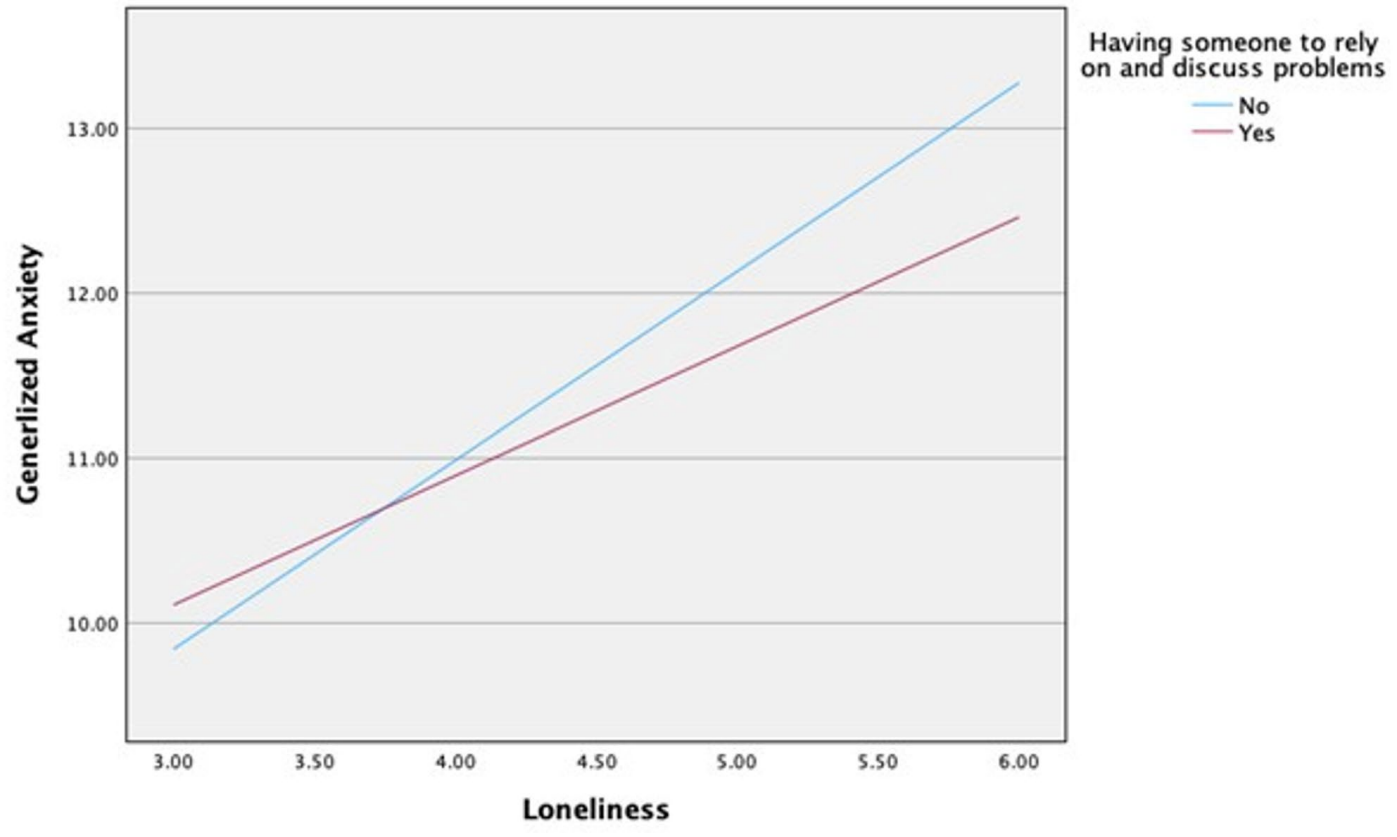
Moderating effect of having someone to rely on for loneliness and generalized anxiety

**Fig. 2:**
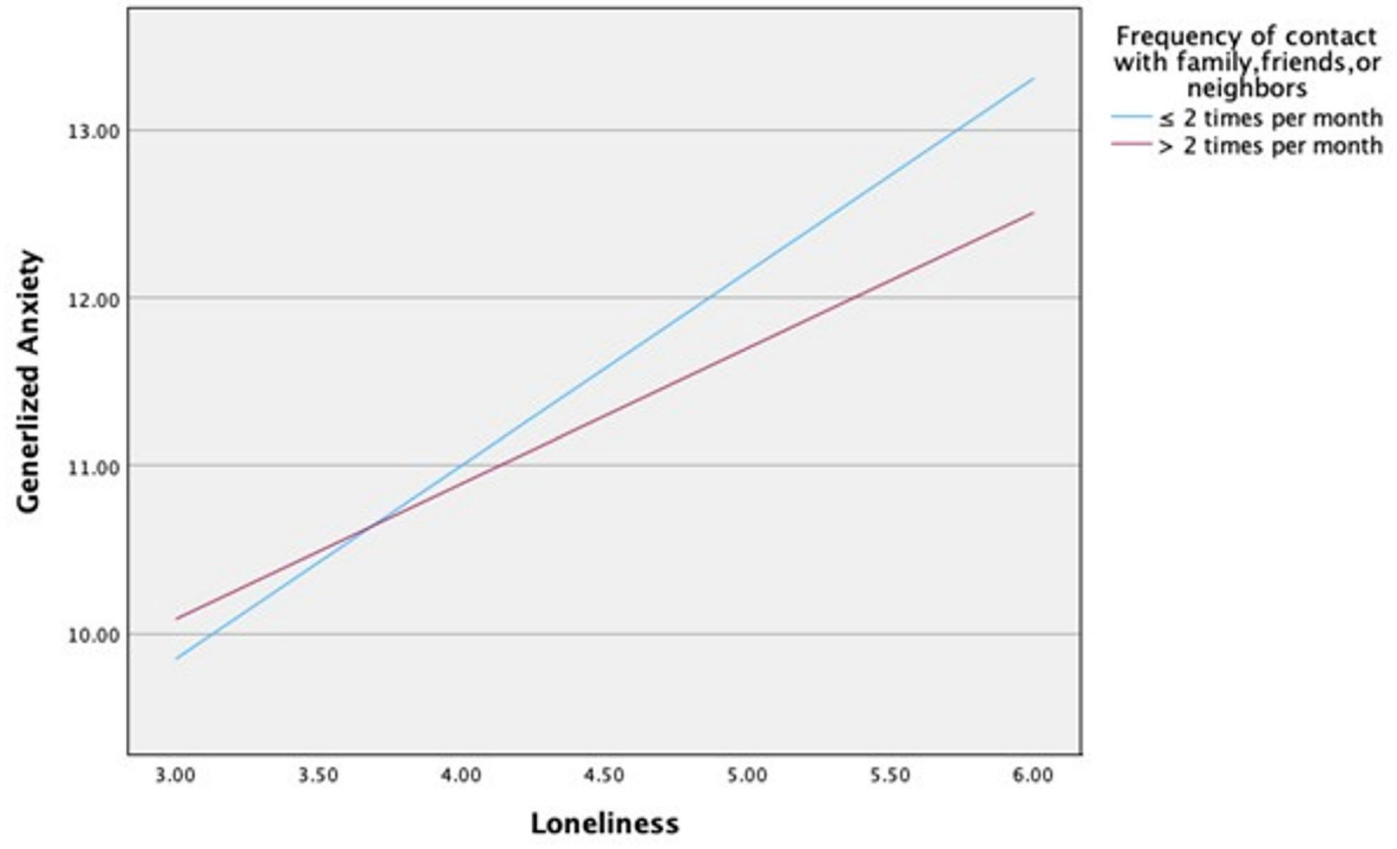
Moderating effect of frequency of contact with others on loneliness and generalized anxiety

Table 4 presents the results of the moderation analyses examining social factors in the association between loneliness and generalized anxiety. Loneliness was significantly associated with generalized anxiety (*p* < 0.001). Having someone to rely on and discuss problems with showed a significant interaction effect on the association between loneliness and generalized anxiety (*b* = − 0.361, 95% CI = − 0.640, − 0.082, t=-2.541, *p* = 0.011). Participants who reported not having someone to rely on and discuss problems with experienced a greater impact of loneliness on generalized anxiety (*b* = 1.144, 95% CI = 0.905, 1.383, t = 9.392, *p* < 0.001) compared with those who reported having such support (*b*= 0.783, 95% CI = 0.638, 0.928, t = 10.606, *p* < 0.001; Fig. 1). Furthermore, frequency of contact with family members, friends, and neighbors showed a significant interaction effect on the association between loneliness and generalized anxiety (*b* = − 0.346, 95% CI = − 0.640, − 0.052, t=-2.309, *p* = 0.021). Among participants who reported a low frequency of social contact (≤ 2 times per month), the impact of loneliness on generalized anxiety was greater (*b* = 1.153, 95% CI = 0.892, 1.413, t = 8.684, *p* < 0.001) than it was among those who reported more frequent contact (> 2 times per month); (*b*= 0.807, 95% CI = 0.667, 0.946, t = 11.335, *p* < 0.001; Fig. 2).

**Table 4:** Moderating effect of the social factors between loneliness and generalized anxiety (*N* = 1084)

Variables	B	Standard errors SE	t	*p*-value	95% CI		R2	∆R2	f^2^
					Lower	Upper			
Loneliness	0.436	0.438	0.997	0.318	− 0.422	1.296			
Living arrangement	−1,881	1.304	−1.442	0.149	−4.440	0.678			
Interaction loneliness by living arrangement	0.246	0.226	1.087	0.277	− 0.198	0.690	0.18	0.001	0.001
Loneliness	0.789	0.190	4.145	< 0.001	0.415	1.163			
Number of close friends and family members	− 0.366	0.613	− 0.596	0.550	−1.570	0.837			
Interaction loneliness by number of close friends and family members	0.088	0.125	0.701	0.483	− 0.158	0.334	0.18	0.000	0.000
Loneliness	1.505	0.254	5.921	< 0.001	1.006	2.004			
Having someone to rely on and discuss problems	1.351	0.738	1.830	0.067	− 0.097	2.800			
Interaction loneliness by having someone to rely on and discuss problems	− 0.361	0.142	-2.541	0.011	− 0.640	− 0.082	0.19	0.006	0.007
Loneliness	1.499	0.274	5.470	< 0.001	0.961	2.037			
Frequency of contact with family members, friends, and neighbors per month	1.276	0.779	1.636	0.102	− 0.253	2.806			
Interaction loneliness by frequency of contact with family members, friends, and neighbors per month	− 0.346	0.149	−2.309	0.021	− 0.640	− 0.052	0.19	0.005	0.006
Loneliness	1.163	0.186	6.228	< 0.001	0.796	1.529			
Volunteering in social activities	0.636	0.621	1.024	0.305	− 0.582	1.855			
Interaction loneliness by volunteering in social activities	− 0.180	0.127	-1.413	0.157	− 0.430	0.070	0.18	0.001	0.001

Note.*p < 0.05* = statistically significant; f^2^= Cohen’s effect size

## Discussion

This study examined the association between loneliness and generalized anxiety and identified potential social factors that moderated this association. With respect to the first research question, the results revealed a positive association between loneliness and generalized anxiety. This finding is consistent with prior international studies [4, 18] and aligns with the loneliness regulatory loop model [7]. According to this model, individuals who experience loneliness may develop negative thoughts and heightened vigilance toward social threats, leading to a self-reinforcing loneliness loop accompanied by increased anxiety [7]. Regarding the second research question, the findings indicated that participants with chronic disorders exhibited higher mean loneliness scores than those without chronic disorders. These findings suggest that loneliness may be a risk factor for chronic health problems and mortality [1, 7, 9] and may lead to more frequent physician visits [4]. Furthermore, loneliness can trigger neurobiological and behavioral changes that make individuals more vulnerable to poor health outcomes [7]. Given the negative impact of loneliness on both physical and mental health, routine screening in health care settings may help identify individuals at increased risk of loneliness [1].

With respect to social factors, participants with less frequent contact with family members, friends, and neighbors (≤ 2 times per month) exhibited higher mean scores for both loneliness and generalized anxiety compared with those with more frequent contact (> 2 times per month). This finding supports previous studies showing that individuals with more frequent social contact are less likely to experience loneliness [5, 9]. In addition, participants who reported having someone to rely on and discuss problems with exhibited lower mean scores for loneliness and generalized anxiety compared with those who did not. This is an expected finding, as the availability of reliable social support plays an important role in enhancing individuals’ ability to cope with stressors and promoting mental well-being [25]. This result is consistent with previous studies reporting associations between social support and loneliness [9] and between social support and anxiety [26]. Another notable finding was that participants who lived alone exhibited higher mean scores for loneliness and generalized anxiety compared with those who lived with others. This finding is consistent with previous research reporting associations between living alone and loneliness [9, 12], as well as between living alone and generalized anxiety [27]. However, cultural context is important when interpreting these findings. The experience of loneliness may be perceived differently across cultures [9]. In Saudi Arabia, social relationships—particularly among family members—are highly emphasized [28, 29]. Individuals often depend on their social networks, especially family members, and expect frequent communication and availability for support. When these expectations are unmet, individuals may experience intense distress [9].

Regarding the third research question, the moderation analysis showed that having someone to rely on and discuss problems with moderated the relationship between loneliness and generalized anxiety. Participants experiencing greater loneliness who lacked someone to rely on and discuss problems with exhibited higher generalized anxiety scores than those who reported having such support. Feeling unsupported by others may exacerbate the emotional distress associated with loneliness, leading to greater anxiety. This finding is consistent with previous studies showing that social support moderates the relationship between loneliness and well-being, including the relationship between depression and loneliness among older adults [19] and between loneliness and personal well-being among young adults [11]. In addition, frequency of contact with family members, friends, or neighbors moderated the association between loneliness and generalized anxiety. Specifically, participants with higher loneliness scores and less frequent contact (≤ 2 times per month) reported higher generalized anxiety scores than those with more frequent contact (> 2 times per month). This finding is consistent with previous research showing that communication with neighbors moderated the association between loneliness and personal well-being [11].

This study has several limitations. First, the cross-sectional design does not allow for causal inferences. Second, self-selection bias may have occurred owing to the use of convenience sampling, which may limit the generalizability of the findings. In addition, social desirability bias may have influenced responses, even though no personal identifying data were collected. Third, although several social factors were examined—such as living arrangements, frequency of contact with others, perceived social support, and participation in social activities—future studies should consider additional factors, including environmental characteristics such as access to green spaces and availability of facilities [9]. Fourth, this study did not assess other factors that may influence anxiety, such as religious beliefs and cognitive functioning. Previous research suggests that these variables may affect mental health; for example, studies among older adults in Greece have reported negative associations between anxiety and religious beliefs [30] and between anxiety and cognitive functioning [31]. Future research should explore these factors to further clarify their role in mental health outcomes.

Furthermore, the generalizability of the findings may be limited and should be interpreted with caution, as males and some regions were underrepresented in the sample compared with Saudi Arabia’s 2022 census data [32]. Although statistically significant moderation effects were observed for frequency of contact with others and perceived social support, these findings should be interpreted cautiously due to the small effect sizes. This is consistent with previous research indicating that interaction effects in psychological research typically yield small effect sizes, with an average f^2^ of 0.009 [33]. Despite the small effect sizes, these findings remain valuable, as they highlight the moderating role of perceived social support and frequency of social contact in the relationship between loneliness and generalized anxiety within the Saudi Arabian context.

Overall, this study provides valuable insights into the association between loneliness and generalized anxiety and the potential moderators of this relationship in Saudi Arabia. It also contributes to the cross-cultural generalizability of findings on the relationship between loneliness and psychopathological conditions, as well as the role of social moderators. The results suggest that this relationship persists even though loneliness may be perceived differently in Saudi Arabia compared with more individualistic Western societies.

## Implications

These results have important implications for health practitioners and policymakers seeking to reduce the risk of loneliness and mitigate its negative health consequences. One key intervention is the implementation of routine screening in health care settings to identify individuals at increased risk of loneliness [1]. In addition, efforts should focus on enhancing social connections within communities by organizing peer support groups and group-based activities, such as sports events, religious gatherings, concerts, and cooking classes [1]. Furthermore, psychological interventions, such as cognitive behavioral therapy (CBT), have been shown to be effective in modifying cognitive biases related to social interaction [7]. Educational programs that address negative thoughts surrounding social interaction may also help mitigate loneliness in the general population [1].

## Conclusion

This study assessed the association between loneliness and generalized anxiety and identified potential social factors that moderated this association among adults in Saudi Arabia. Loneliness was significantly associated with generalized anxiety. The moderation analyses indicated that both frequency of contact with others and having someone to rely on and discuss problems with moderated this relationship. Mental health professionals and policymakers should develop appropriate interventions and public health policies to alleviate the negative health consequences of loneliness. Future research, particularly in Saudi Arabia, should examine other potential moderators of the association between loneliness and mental health.

## Data Availability

The datasets used and analyzed during the current study are available from the corresponding author on reasonable request.

## Abbreviations

COVID-19: Coronavirus disease
GAD-7: Generalized Anxiety Disorder-7
UCLA: University of California, Los Angeles
CBT: Cognitive behavioral therapy
